# *Streptomyces nigra* sp. nov. Is a Novel Actinobacterium Isolated From Mangrove Soil and Exerts a Potent Antitumor Activity *in Vitro*

**DOI:** 10.3389/fmicb.2018.01587

**Published:** 2018-07-18

**Authors:** Can Chen, Yanghui Ye, Ruijun Wang, Yinglao Zhang, Chen Wu, Sanjit C. Debnath, Zhongjun Ma, Jidong Wang, Min Wu

**Affiliations:** ^1^Laboratory of Marine Microbial Resources Utilization, Ocean College, Institute of Marine Biology, Zhejiang University, Hangzhou, China; ^2^Biomedical Research Program, School of Life Sciences, Anhui Agricultural University, Hefei, China; ^3^Institute of Hydraulic and Marine Engineering, School of Hydraulic and Environmental Engineering, Zhejiang University of Water Resources and Electric Power, Hangzhou, China; ^4^Department of New Drug Screening, Zhejiang Hisun Pharmaceutical Co., Ltd., Taizhou, China

**Keywords:** *Streptomyces nigr*a sp. nov., polyphasic taxonomy, antitumor, bioactive metabolites, diketopiperazine

## Abstract

A new bacterial strain, designated 452^T^, was isolated from the rhizosphere soil of the mangrove *Avicennia marina* in China. As determined, its cell wall peptidoglycan contained LL-diaminopimelic acid; MK-9(H8) and MK-9(H6) were the major isoprenoid quinones; and iso-C_16:0_ (31.3%), anteiso-C_15:0_ (16.9%), and iso-C_15:0_ (12.5%) were the major cellular fatty acids (>10.0%). Phylogenetic analysis based on the 16S rRNA gene sequence revealed that strain 452^T^ formed a distinct lineage in the clade of the genus *Streptomyces*, and was closely related to *S. coerulescens* DSM 40146^T^ (99.6% sequence identity), *S. bellus* DSM 40185^T^ (99.5%), and *S. coeruleorubidus* DSM 41172^T^ (99.3%). The DNA-DNA relatedness between strain 452^T^ and these type strains ranged between 29.3 and 42.3%. Based on the phenotypic, chemotaxonomic, and phylogenetic features, the strain 452^T^ is considered to represent a novel species of the genus *Streptomyces*, for which the name *Streptomyces nigra* sp. nov. is proposed. The type strain is 452^T^ (=KCTC 39960^T^ = MCCC 1K03346^T^). Further, strain 452^T^ extracts exhibited a pronounced antitumor activity against human cancer cell lines A549, HCT-116, and HepG2, but not against normal human colon cells CCD-18Co. Active substances in the fermentation broth of strain 452^T^ were isolated by bioassay-guided analysis, and then purified using a macroporous resin, silica gel, sephadex LX-20 column, and semi-preparative high-performance liquid chromatography (HPLC). Eight proline-containing diketopiperazines, namely, cyclo(Pro-Ala), cyclo(Pro-Gly), cyclo(Pro-Phe), cyclo(Pro-Met), cyclo(Pro-Val), cyclo(Pro-Leu), cyclo(Pro-Tyr), and cyclo(L-Leu-trans-4-hydroxy-L-Pro), were identified by electrospray ionization mass spectrometry (MS) and nuclear magnetic resonance (NMR). The compounds displayed different levels of cytotoxicity. The highest cytotoxicity was exhibited by cyclo(Pro-Ala) and cyclo(Pro-Met) against A549 cells, and cyclo(Phe-Pro) and cyclo(Pro-Ala) against HCT-116 cells, with average IC_50_ values equal to 18.5, 27.3, 32.3, and 47.6 μg/mL, respectively. The diversity of diketopiperazines and other chemicals produced by 452^T^ was further investigated using gas chromatography (GC)-MS and liquid chromatography (LC)-MS. The analysis revealed 16 types of metabolites with antitumor activity and 16 other types of diketopiperazines. Hence, extracts of the newly identified strain may be used a starting material for the development of antitumor agents.

## Introduction

The genus *Streptomyces* contains over 770 species with valid published names at the time of writing^1^. Numerous species from this genus produce antibiotics and several other biologically important compounds, including herbicides, antiparasitic agents, immunosuppressants, and other compounds of industrial interest (Běhal, 2000; Chater, 2006; Bull and Stach, 2007; Schinke et al., 2017; Ser et al., 2017). *Streptomyces* are widely distributed in terrestrial ecosystems, especially in the soil. Specific marine environments are pronouncedly different from the terrene environments, and stimulate the production of novel metabolites by marine microorganisms and the evolution of countless new microbes (Simister et al., 2016; Dudek et al., 2017; Kamjam et al., 2017; Petro et al., 2017; Picard, 2017). Marine environments are considered to be a potential source for the discovery of novel *Streptomyces* that produce bioactive compounds (Sunagawa et al., 2015; Christensen and Martin, 2017; Wei et al., 2018). In recent years, over 20 novel species of the genus *Streptomyces* have been identified in marine and mangrove environments^2^. The mangrove occupies millions of hectares of coastal areas; it is one of the most dynamic environments in the world, and the habitat of various flora and fauna of terrestrial, freshwater, and marine species (Law et al., 2017). Recently, utilization of the mangrove microorganism resource has received a lot of attention, subsequently leading to the discovery of novel *Streptomyces* species (Hong et al., 2009; Lee et al., 2014).

The genus *Streptomyces* is an important biological resource for the exploration and discovery of antitumor substances. Anthracycline-based drugs, e.g., erythromycin, doxorubicin, and epirubicin, are widely used in the clinic to treat various types of cancer, including breast cancer, small cell lung cancer, cervical cancer, and head and neck cancer. These drugs are produced by *S. peucetius* and *S. nogalater* (Nobili et al., 2009; Zhang Z.G. et al., 2015; Lu et al., 2017). The polypeptide metabolite dactinomycin is an antibiotic with antibacterial and antifungal activity. Its antitumor activity was confirmed by Juliano and Stamp (1978). Similarly, bleomycin, a natural hybrid peptide-polyketide metabolite and an antitumor drug, is produced by *S. plicatus*, *S. chrysomallus*, and *S. verticillus* (Katz et al., 1961; Juliano and Stamp, 1978; Du et al., 2000; Lam et al., 2002; Cai et al., 2016; Lee J.H. et al., 2016). Mithramycin is an aromatic polyketide that contains two deoxysugar chains (a disaccharide of two D-olivoses, and a trisaccharide consisting of D-olivose), a D-oliose, and a D-mycarose, which inhibits testicular cancer and is produced by *S. argillaceus* (Fernandez et al., 1998; Bilyk and Luzhetskyy, 2016; Novakova et al., 2018). *Streptomyces* also produce 2,5-diketopiperazines (also known as 2,5-dioxopiperazines or cyclic dipeptides), a class of organic molecules, in which the two nitrogen atoms of the 6-membered ring of piperazine form amide linkages (Huang et al., 2014). They are the minimum natural cyclopeptides and possess important biological activities, such as antitumor, antiviral, antifungal, antibacterial, and antihyperglycemic activities (Martins and Carvalho, 2007; Borthwick and Da Costa, 2017). Remarkably, these bacteria also produce mitomycin C and enediyne compounds (including calicheamicins, neocarzinostatin, and lidamycin), which have unique structural characteristics and display pronounced antitumor-activity (Hata et al., 1956; Mao et al., 1999; Liu et al., 2005; Lee, 2009).

In the current study, we isolated a novel member of the genus *Streptomyces* from the rhizosphere soil of the mangrove *Avicennia marina* in China, evaluated its antitumor activity *in vitro*, and characterized the active compounds in its extract.

## Materials and Methods

### Sample Collection and Strain Isolation

Strain 452^T^ was isolated from a sample of a rhizosphere soil collected during the spring of 2016 in the mangrove *Avicennia marina* forest of Zhangzhou (24°20’, 117°45’), Fujian Province (China), and stored at 4°C until use. The soil sample were suspended in sterile water and diluted in a tenfold series. The dilutions were then spread onto modified ZoBell 2216E agar plates and incubated at 28°C for 5 days. After incubation, a brown colony that secreted dark brown pigment was selected and purified by repeated streaking onto marine agar 2216 (MA; Difco). The strain was named 452^T^. It was routinely cultured on MA and preserved at –80°C in marine broth 2,216 (MB; Difco) supplemented with 20% (v/v) glycerol.

### Genomic and Phylogenetic Analyses

Genomic DNA was extracted for amplification in a polymerase chain reaction as described by Hong et al. (2009). The DNA G + C content was determined by reversed-phase high-performance liquid chromatography (HPLC) (Mesbah et al., 1989) using the genomic DNA of *Escherichia coli* K-12 and salmon sperm DNA (Sigma) as calibration standards. The 16S rRNA gene of strain 452^T^ was amplified using the universal primers 27F (5’-AGAGTTTGATCMTGGCTCAG-3’) and 1492R (5’-TACGGYTACCTTGTTACGACTT-3’) (Weisburg et al., 1991). The amplification products were cloned into the pMD19-T vector (TaKaRa) and sequenced. The determined 16S rRNA gene sequence (1,490 bases) was analyzed in pairwise sequence alignments using the BLASTN program^3^ and the EzTaxon-e server^4^ (Yoon et al., 2017). Multiple sequence alignments based on the 16S rRNA gene sequences of strain 452^T^ and related taxa were done as described by Zhang X.Q. et al. (2015) and Zhang Z.G. et al. (2015) using MEGA program version 5 (Tamura et al., 2011). Phylogenetic trees were reconstructed by using the neighbor-joining, maximum-likelihood, and maximum-parsimony methods based on 1,000 replications, and bootstrap analysis was performed.

Genomic DNA extraction for DNA-DNA hybridization of strain 452^T^, *S. coerulescens* DSM 40146^T^, *S. bellus* DSM 40185^T^, and *S. coeruleorubidus* DSM 41172^T^ (obtained from the DSMZ; unless otherwise stated, all strains were incubated in MA or MB at 28°C) were performed following a protocol of Cashion et al. (1977). DNA-DNA hybridization values of strain 452^T^ and type strains were determined by using a Beckman DU800 spectrophotometer according to the method of Zhang et al. (2010). The hybridization temperature was set at 57°C. The complete genome of strain 452^T^ was sequenced and assembled by Beijing Genomics Institute (BGI, Beijing, China), and the genome sequences of reference strains were retrieved from the GenBank database. The average nucleotide identity (ANI) was calculated using the OrthoANIu algorithm of the Chun lab’s online Average Nucleotide Identity calculator (Lee I. et al., 2016). *In silico* DNA-DNA hybridization (DDH) values were calculated by genome-to-genome distance calculator (GGDC) (Meier-Kolthoff et al., 2013).

### Phenotypic Characterization

Growth of strain 452^T^ was tested in the presence of various NaCl concentrations (0–15.0%, w/v, at 0.5% increments) in MB (pH 7.0). The growth temperature range was tested at 4, 10, 15, 20, 25, 28, 30, 35, 40, 45, and 50°C in MB (pH 7.0). The growth pH range was determined at 0.5-pH unit intervals by supplementing MB with 30 mM buffering agents at 28°C: 2-(*N*-morpholino)ethanesulfonic acid (pH 5.5–6.4), 3-(*N*-morpholino)propanesulfonic acid (pH 6.5–7.9), tricine (pH 8.0–8.9), and bis-Tris propane (pH 9.0–9.5) (Zhong et al., 2014). The optimal growth was determined after 5 days of incubation, and the growth limits were assessed after 14 days of incubation (Zhang X.Q. et al., 2015). Strain 452^T^ was incubated at 28°C for 5 days on MA medium, and cell morphology was examined and observed by transmission electron microscopy (80 kV, JEM-1230; Jeol) after uranyl acetate (0.5%, w/v) staining, scanning electron microscope (3.0 kV, SU8010, Hitachi) fixation by osmium tetroxide vapor (4%, w/v), and optical microscopy (BX40; Olympus) after gram staining. The culture characteristics were determined following growth on ISP 2, ISP 3, ISP 4, ISP 5, ISP 6, and ISP 7 agars (Shirling and Gottlieb, 1966); *Streptomyces* agar (Atlas, 2010), starch casein nitrate agar (Ser et al., 2016b), nutrient agar (Odds, 1981), potato-glucose agar (Wang et al., 2017), tryptone soya agar (Williams et al., 1983), and MA 2216 (Difco) for 14 days at 28°C. The colors of substrate, aerial mycelium and any soluble pigments produced were determined by comparison with chips from the ISCC-NBS color charts (Kelly and Judd, 1964).

Degradation of starch; oxidase and catalase activities; hydrolysis of Tweens 20, 40, 60, 80, hypoxanthine, and xanthine; nitrate reduction and urease activity; the ability to hydrolyze casein, chitin, carboxymethyl (CM) cellulose, filter paper, and gelatin; H_2_S production; and methyl red and Voges-Proskauer reactions were all evaluated as described previously (Chen et al., 2017). Degradation of tyrosine was determined on MA supplemented with 5 g/L tyrosine. The presence of flexirubin-type pigments was investigated using 20% (w/v) KOH solution. Anaerobic growth was determined in an anaerobic system (AnaeroPack-MicroAero, 2.5-L, MGC, Japan) on MA supplemented with 20 mM sodium thiosulfate, 5 mM sodium sulfite, 20 mM sodium sulfate, 5 mM sodium nitrite, or 20 mM sodium nitrate as electron acceptors (Zhong et al., 2016). Utilization of carbon substrates (0.5%, w/v) was tested according to the protocol of Dong and Cai (2001) using whole components of the inorganic salts of MB with yeast extract (0.01%, w/v) as growth factors. Acid production was evaluated using the marine oxidation-fermentation medium supplemented with 1% (w/v) sugars (Wu et al., 2017). Enzyme activities, and other physiological and biochemical traits were analyzed using API ZYM and API 20NE stripes (bioMérieux) according to the manufacturer’s instructions. The three reference strains were used as controls in the above tests.

### Chemotaxonomic Characterization

Strain 452^T^, *S. coerulescens* DSM 40146^T^, *S. bellus* DSM 40185^T^, and *S. coeruleorubidus* DSM 41172^T^ cells were harvested during the third quadrants on MA (28°C, 3 days) for cellular fatty acid analysis. Whole-cell fatty acids were analyzed according to the instructions of the Microbial Identification System (MIDI; Microbial ID) using the standard MIS library generation software version 4.5. Isoprenoid quinones were extracted with a chloroform/methanol mixture (2:1, v/v) from freeze-dried cells (500 mg) and analyzed by LC-MS (Agilent) (Komagata and Suzuki, 1987). Polar lipids were extracted with a chloroform/methanol mixture (1:2, v/v) and identified by two-dimensional thin-layer chromatography on silica gel 60 F_254_ plates (Merck). Molybdophosphoric acid, ninhydrin reagent, molybdenum blue, and α-naphthol/H_2_SO_4_ reagents were sprayed onto the plates to detect total lipids, lipids containing free aminolipids, phosphorus-containing lipids, and glycolipids, respectively (Tindall, 1990).

### Preparation of the 452^T^ Fermented Broth and Extract

Strain 452^T^ was inoculated into a 500 mL Erlenmeyer flask containing 200 mL of tryptone soya liquid medium and incubated at 28°C for 7 days on a rotary shaker at 150 rpm, as seed medium prior to the fermentation process. Then, 1% (v/v) of the starting inoculum was transferred into 1-L Erlenmeyer flasks containing 600 mL of MB, and the incubation was continued on a rotary shaker at 180 rpm at 30°C for 10 days. The cell-free supernatant was collected after centrifugation at 13,000 ×*g* for 10 min, freeze-dried, and extracted repeatedly with methanol. The organic phase was separated and evaporated to dryness using a rotary evaporator at 40°C (Ahmad et al., 2017). The extracted residues were suspended in dimethyl sulfoxide for bioactivity, gas chromatography-mass spectrometry (GC-MS), and liquid chromatography (LC)-MS assays.

### *In Vitro* Antitumor Cytotoxicity Testing

HCT-116 (human colorectal carcinoma), A549 (human lung carcinoma), SF-268 (human central nervous system cancer), HepG2 (human hepatocellular carcinoma), and CCD-18Co (human normal colon cells) cell lines were obtained from the Department of New Drug Screening, Zhejiang Hisun Pharmaceutical Co., Ltd. (Taizhou, China). U87 (human glioblastoma cell) and MCF-7 (human breast adenocarcinoma cell) cells were provided by the School of Pharmacy of the China Pharmaceutical University (Nanjing, China). All cell lines used in this study were maintained in Dulbecco’s modified Eagle’s medium supplemented with 10% (w/v) fetal bovine serum in a humidified incubator (5% CO_2_ in air at 37°C). The antitumor activity of 452^T^ extracts with different concentrations (20, 100, or 200 μg/mL) were investigated by the 3-(4,5-dimethylthiazol-2yl)-2,5-diphenyltetrazolium-bromide assay, doxorubicin was tested as positive control, and MB with same preparation method of 452^T^ extract was tested as negative control. Briefly, confluent cells were harvested and seeded at a density of 6 × 10^4^ cells/well into a sterile flat bottom 96-well plate overnight, the cells were treated with different concentrations of the extracts for 48 h and growth inhibition was measured by determining the optical density at 570 nm, and the assay was performed according to an established method (Skehan et al., 1990). The cell morphology was observed by inverted microscope (Nikon TS100).

### GC-MS and LC-MS Analyses

GC-MS analysis was performed using an Agilent 7890 gas chromatograph system coupled with an Agilent 5975C mass spectrometer. The system utilized DB-Wax (30 m × 250 μm inner diameter, 0.25 μm film thickness). For the analysis, a 1 μL aliquot of the analyte was injected in the splitless mode. Helium was used as the carrier gas, the front inlet purge flow was 3 mL/min, and the gas flow rate through the column was 1 mL/min. The initial temperature was kept at 40°C for 5 min, then raised to 250°C at a rate of 5°C/min, and finally maintained at 250°C for 5 min. The energy was -70 eV in the electron impact mode. The MS data were acquired in a full-scan mode over 20–400 m/*z*. The extracted constituents were identified by comparing the MS data with data from the NIST 05 Spectral Library.

LC-MS/MS analyses were performed at the Zhejiang Hisun Pharmaceutical Co., Ltd. The MS spectra were acquired using an LC-MS/MS ion trap time-of-flight (IT-TOF) spectrometer (Shimadzu, Japan) equipped with an electro-spray ionization source (Abe et al., 2005) operated in the positive and negative ion modes. HPLC was performed according to Zhou et al. (2015). Briefly, the sample (2 μL) was injected onto a column (Waters ACQUITY UPLC BEH Amide, 2.1 × 100 mm, 1.7 μm) and resolved at a flow rate of 0.2 mL/min. Mobile phases used were: A (0.1%, v/v, formic acid/water) and B (0.1%, v/v, formic acid/acetonitrile). The separation gradient was as follows: 0–30 min: 10–100% B.

### Isolation and Characterization of Bioactive Metabolites

The procedure of cultivation in method 2.5 was repeated, filtrate (15 L) of the culture broth was collected. The filtrate was separated and purified on an HP-20 macroporous resin (Mitsubishi, Japan) column, and eluted with absolute ethyl alcohol. After evaporation of the menstruum *in vacuo*, the residue (5.2 g) was resolved by chromatography on a silica gel column eluted with CHCl_3_/MeOH mixtures with a growing polarity (100:0–30:70, v/v) to obtain six fractions (F1–6). Bioactivity assays (*in vitro* antitumor toxicity) indicated that F4 (CHCl_3_/MeOH, 50:50) and F5 (CHCl_3_/MeOH, 40:60) fractions were cytotoxic *in vitro*. The active fractions (F4 and 5) were repeatedly purified, and separated on Sephadex LH-20 (MeOH) and using semi-preparative HPLC (Agilent 1100, Zorbax SB-C18, 5 μm, 250 × 9.4 mm inner diameter; Agilent, Palo Alto, CA, United States) to obtain compounds **1–8** (5.1–10.7 mg of each).

Structural identification of the bioactive metabolites was based on spectroscopic analysis. ^1^H nuclear magnetic resonance (NMR) and ^13^C NMR spectra were acquired with a Bruker DRX-400 spectrometer (400 MHz for ^1^H and 100 MHz for ^13^C) (Bruker, Rheinstetten, Germany). Chemical shifts are reported in ppm. (δ), using residual CHCl_3_ (δ_H_ 7.26 ppm; δ_C_ 77.0) as an internal standard, with coupling constants (J) in Hz.^1^H and ^13^C NMR assignments were supported by ^1^H-1H COSY, HMQC, and HMBC experiments. The electrospray ionization MS data were recorded using the Bruker APEX III 7.0T spectrometer.

## Results and Discussion

### Genomic and Phylogenetic Analyses

**FIGURE 1:**
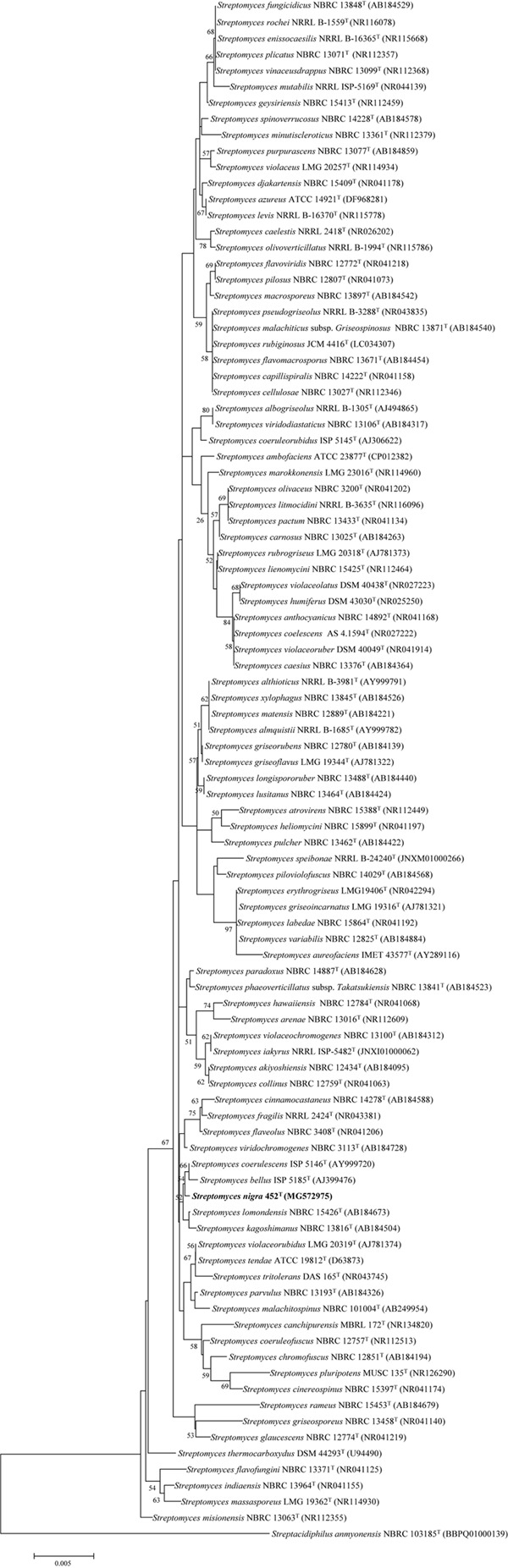
Neighbor-joining phylogenetic tree based on the 16S rRNA gene sequences of strain 452^T^ and representatives of related taxa. Bootstrap values were expressed as a percentage of 1,000 replicates and only values >50% are shown at the branch points. Bar, 0.005 substitutions per nucleotide position.

The 16S rRNA gene sequence of strain 452^T^ comprised 1490 bases. According to the information in the EzTaxon server, it was most closely related to *S. coerulescens* DSM 40146^T^ (99.6%), and shared 99.6–98.5% sequence similarity with the type strains of other species of the genus *Streptomyces*. The 16S rRNA gene sequences of strain 452^T^ and the type strains of other genera shared <90% similarity. Phylogenetic trees were reconstructed from 1000 replicates each for bootstrap analysis using the neighbor-joining (**Figure 1**), maximum-parsimony (Supplementary Figure S1), and maximum-likelihood (Supplementary Figure S2) methods. These analyses revealed that strain 452^T^ belonged to a clade of the genus *Streptomyces* and formed a distinct lineage among the most closely related species: *S. coerulescens* DSM 40146^T^ (99.6%), *S. bellus* DSM 40185^T^ (99.5%), and *S. coeruleorubidus* DSM 41172^T^ (99.3%). Hence, these three type strains were obtained from DSMZ (Germany), and selected as reference strains in the current study, unless otherwise stated, all strains were incubated in MB at 28°C. Twenty-five additional strains were chosen as reference strains which were representatives of adjacent clusters from the NJ, MP, and ML trees. Data of these strains were collected from literatures and compared with strain 452^T^ (Supplementary Table S1). The DNA G + C content of strain 452^T^ was 71.7 mol%, which is similar to other strains from the *Streptomyces* genus. The DNA-DNA relatedness values between strain 452^T^ and *S. coerulescens* DSM 40146^T^ (42.3 ± 1.0%), *S. bellus* DSM 40185^T^ (29.3 ± 0.4%), and *S. coeruleorubidus* DSM 41172^T^ (37.8 ± 0.7%) were well below the threshold value (70%) for determining a bacterial species (Wayne et al., 1987). The whole Genome Shotgun project of strain 452^T^ has been deposited at DDBJ/ENA/GenBank under the accession CP029043. The ANI values and *in silico* DDH values between strain 452^T^ and reference strains were 71.16–84.86% and 15.27–22.18%, respectively (**Table 1**). Which were below the 95% threshold value for ANI, 70% for GGDC proposed for the delineation of bacterial species, indicating that strain 452^T^ belongs to a novel species of the genus *Streptomyces* (Richter and Rossello-Mora, 2009; Auch et al., 2010).

**Table 1:** Average nucleotide identity (ANI) and *in silico* DNA-DNA hybridization (DDH) values between strain 452^T^ and the reference type strains of related species of genus *Streptomyces.*

Strains	Strain 452^T^	
	
	ANI(%)	DDH(%)
Strain 452^T^	100	0.00
*Streptomyces almquistii* NRRL B-1685^T^ (LIPO00000000)	72.81	19.84
*Streptomyces azureus* ATCC 14921^T^ (BBYS00000000)	83.86	14.72
*Streptomyces caelestis* NRRL 2418^T^ (LGCN00000000)	83.37	16.32
*Streptomyces cellulosae* NBRC 13027^T^ (JOEV00000000)	79.00	16.17
*Streptomyces flaveolus* NBRC 3408^T^ (JNWV00000000)	84.25	16.62
*Streptomyces glaucescens* NBRC 12774^T^ (MUND00000000)	82.80	16.53
*Streptomyces minutiscleroticus* NBRC 13361^T^ (MAZZ00000000)	84.86	15.73
*Streptomyces olivaceus* NBRC 3200^T^ (JOFH00000000)	79.37	18.12
*Streptomyces pactum* NBRC 13433^T^ (LIQD00000000)	83.62	16.51
*Streptomyces parvulus* NBRC 13193^T^ (NZ CP015866)	82.92	17.32
*Streptomyces pluripotens* MUSC 135^T^ (NZ CP021080)	81.37	18.66
*Streptomyces speibonae* NRRL B-24240^T^ (JNXM00000000)	84.05	15.27
*Streptomyces violaceoruber* DSM 40049^T^ (JOCE00000000)	75.35	19.31
*Streptomyces viridochromogenes* NBRC 3113^T^ (ACEZ00000000)	83.37	16.55
*Streptomyces violaceorubidus* LMG 20319^T^ (JODM00000000)	84.30	16.83
*Streptomyces rochei* NRRL B-1559^T^ (MUMD00000000)	71.16	22.18
*Streptomyces ambofaciens* ATCC 23877^T^ (NZ CP012382)	83.05	16.45
*Streptomyces collinus* NBRC 12759^T^ (NC CP021985)	82.95	17.26
*Streptomyces griseoflavus* LMG 19344^T^ (LGUW00000000)	74.15	21.07
*Streptomyces griseorubens* NBRC 12780^T^ (JJMG00000000)	82.79	16.40
*Streptomyces iakyrus* NRRL ISP-5482^T^ (JNXI00000000)	80.17	18.56
*Streptomyces misionensis* NBRC 13063^T^ (FNTD00000000)	82.27	17.26
*Streptomyces pseudogriseolus* NRRL B-3288^T^ (MUNG00000000)	79.50	15.71
*Streptomyces violaceus* LMG 20257^T^ (JNXN00000000)	72.89	17.45
*Streptomyces xylophagus* NBRC 13845^T^ (JNWO00000000)	79.33	16.83

### Phenotypic Characterization of Strain 452^T^

Strain 452^T^ grew well on ISP 2, ISP 3, ISP 4, ISP 5, ISP 7, MA, and tryptone soya agars after 7–14 days at 28°C; it grew moderately well on ISP 6 agar, *Streptomyces* agar, starch casein nitrate agar, nutrient agar, and potato-glucose agar, which was distinct from reference strains (Supplementary Table S2). As shown in Supplementary Figure S3, the strain formed a branched substrate mycelium, sparse aerial hyphae that differentiated into straight to flexuous chains and smooth bud-shaped spores on the ends of the aerial mycelium were formed on MA. The colors of the aerial, substrate mycelium, diffused pigment, and spore surface were media-dependent and are shown in **Table 2** and Supplementary Table S2; these characteristics distinguished this strain from the type strains. Strain 452^T^ was positive for catalase activity; hydrolysis of casein, starch, nitrate, and gelatin; and production of H_2_S. In contrast to the reference strains, for examples, *S. bellus* DSM 40185^T^ and *S. coeruleorubidus* DSM 41172^T^ were negative for catalase; *S. coerulescens* DSM 40146^T^ was positive for oxidase, *S. bellus* DSM 40185^T^ was positive for anaerobic, which were unique characteristics distinct from other reference strains and 452^T^. All reference strains were negative for production of H_2_S, degradation of casein and gelatin, utilization of D-fructose as sole carbon, which were positive for strain 452^T^. All reference strains were positive for α-chymotrypsin and able to utilized xylitol as sole energy source which were not shown in strain 452^T^. Additionally, large numbers of differential features existed between strain 452^T^ and reference strains which shown in Supplementary Table S1, indicating that strain 452^T^ cannot be affiliated with any related species. In general, based on its phenotypic characteristics, strain 452^T^ could be differentiated from the closely related type strains; these properties were consistent with the assignment of the strain to the genus *Streptomyces*.

**Table 2:** Differential phenotypic characteristics of strain 452^T^ and its most closely related species.

Characteristic	1	2	3	4
ACTIVE OF		
Catalase	+	–	w	–
Oxidase	–	–	+	–
Anaerobic	–	+	–	–
H_2_S production	+	–	–	–
Degradation of:				
Tween 20	–	+	+	–
Tween 80	–	+	–	+
Casein	+	–	–	–
Gelatin	+	–	–	–
Tests of API ZYM		
Esterase lipase (C8)	–	+	–	+
*α*-chymotrypsin	–	+	+	+
*α*-galactosidase	–	w	w	+
*N*-acetyl-*β*-glucosaminidase	+	–	w	–
*α*-mannosidase	+	–	w	w
CARBON UTILIZATION		
Succinic acid	+	–	+	+
D-maltose	+	–	+	+
Sodium citrate	+	–	–	+
D-lactose	+	–	+	+
Iinositol	+	–	+	+
Sucrose	+	–	+	+
D-mannose	+	+	+	–
D-fructose	+	–	–	–
D-galactose	+	+	–	–
L-arginine	+	+	+	–
Xylitol	–	+	+	+
D-melibiose	–	–	+	+
MAJOR FATTY ACIDS (%)		
C_16:0_	6.5	12.1	6.5	8.5
C_18:0_	tr	5.5	1.8	tr
iso-C_15:0_	12.5	15.5	12.4	20.1
iso-C_16:0_	31.3	21.0	14.3	14.1
iso-C_17:0_	2.9	8.6	6.8	5.2
iso-C_16:0_ H	6.1	2.3	5.6	1.9
anteiso-C_15:0_	16.9	9.4	8.2	8.5
Summed feature 9^∗^	3.2	7.8	8.1	11.2
MORPHOLOGY (ON ISP4)		
Color of aerial	Grayish blue	Gray	White	White
MORPHOLOGY (ON ISP5)		
Color of aerial	White	White	–	Pink
Color of substrate	Yellow	Orange	–	Pink
MORPHOLOGY (ON ISP7)		
Color of aerial	Gray	Yellowish white	White	Gray
Color of substrate	Gray	Yellow	White	Gray
Colony surface	Smooth	Rough	Hairy	Hairy
MORPHOLOGY (ON NA)		
Color of aerial	White	Gray	Yellowish white	Gray
Color of substrate	Yellowish brown	White	Yellowish brown	Yellowish brown
MORPHOLOGY (ON MA)		
Color of aerial	Gray	White	Gray	Brown
Color of substrate	Gray	Reddish brown	Gray	Brown
Colony surface	Rough	Hairy	Rough	Smooth

*Taxa: 1, strain 452^*T*^; 2, Streptomyces bellus DSM 40185^*T*^; 3, Streptomyces coerulescens DSM 40146^*T*^; 4, Streptomyces coeruleorubidus DSM 41172^*T*^. All strains were positive for alkaline phosphatase, esterase (C4), leucine arylamidase, valine arylamidase, trypsin, acid phosphatase, naphthol-AS-BI-phosphohydrolase, β-galactosidase, β-glucosidase, and nitrate reduction; degradation of starch; and utilization as a sole carbon: erythritol, D-ribose, D-sorbitol, trehalose, salicylic acid, D-glucose, D-mannitol, L-alanine, glycerine, sodium gluconate, sodium malonate, DL-methionine, and L-tryptophan. All strains were negative for lipase (C14), cysteine arylamidase, β-glucuronidase, α-fucosidase, nitrite reduction; degradation of CM-cellulose, tyrosine, Tween 40, Tween 60, and filter paper; flexirubin-type pigment, motility, methyl red testing, Voges-Proskauer reaction; and utilization of D-arabinose, D-xylose, and L-sorbose as sole carbon sources. -, not detected or no growth; tr, trace amount (<1%). ^∗^Summed feature 9 (ECL 16.443) is composed of C16:0 10-methyl and/or ios-C_*17:1*_ω9c.*

### Chemotaxonomic Characterization of Strain 452^T^

The results of chemotaxonomic analysis revealed that MK-9(H8) (74.1%) and MK-9(H6) (22.2%) were the major respiratory quinones of strain 452^T^. The cell wall of strain 452^T^ contained LL-diaminopimelic acid as the diamino acid, and was hence type-I cell wall (Lechevalier et al., 1970). Glutamic acid, alanine, and aspartic acid were the major amino acids of the cell wall. Whole-cell hydrolysates predominantly contained glucose, and small traces of ribose and mannose. The detailed fatty acid and polar lipid profiles of strain 452^T^ and the reference strains are shown in Supplementary Table S3 and Supplementary Figure S4, respectively. The major fatty acids (>5%) detected in strain 452^T^ included iso-C_16:0_ (31.3%), anteiso-C_15:0_ (16.9%), iso-C_15:0_ (12.5%), C_16:0_ (6.5%), iso-C_16:0_ H (6.1%), anteiso-C_17:0_ (5.7%), iso-C_14:0_ (5.4%), and summed feature 3 (C_16:1_*ω*7*c* and/or C_16:1_*ω*6*c*, 5.2%). The polar lipid profile of strain 452^T^ was comprised of diphosphatidylglycerol, phosphatidylethanolamine, phosphatidylglycerol, phosphatidylinositol, phosphatidylinositol mannoside, three unidentified phospholipids, an unidentified aminolipid, an unidentified phosphoaminolipid and an unidentified lipid. The fatty acid and polar lipid profiles of strain 452^T^ were similar to those of the reference strains (Supplementary Table S3), despite some differences in their proportions and minor constituents, respectively. Chemotaxonomic features of strain 452^T^ were also similar to the features of other species from the genus *Streptomyces* (Lee and Whang, 2016; Ser et al., 2016a; Law et al., 2017; Röttig et al., 2017; Wang et al., 2017).

### Cytotoxic Activity of the Extract From Strain 452^T^

The potential anti-proliferative effect of extract of strain 452^T^ was evaluated using several human-derived cancer cell lines (U87, MCF-7, HCT-116, HepG2, A549, and SF-268) and normal human colon cells (CCD-18Co), which were tested *in vitro* using growth-inhibition assays. The results are summarized in Supplementary Figure S5. All cancer cell lines showed susceptibility to the extract of strain 452^T^ with inhibition ratio of 90.8–60.0% (100 μg/mL of the extract was tested). As indicated, HCT-116 cells were most sensitive to the extract, with the inhibition ratio of 90.8 ± 4.2% and 72.1 ± 2.7% at concentration of 100 μg/mL and 20 μg/mL, respectively. HepG2 and A549 cells were more sensitive (inhibition ratio >80% at 100 μg/mL and >60% at 20 μg/mL) to the extract than the U87, MCF-7 and SF268 cells. The inhibition ratios of U87, MCF-7 and SF268 cells were 80.0–55.0% (100 μg/mL of the extract was tested), and 55.0–40.0% (20 μg/mL of the extract was tested). In contrast, the normal human colon cells CCD-18Co were resistant to the extract, with the inhibition ratio lower than 15%. Morphological changes of all tested cell lines in response to extract of 452^T^ after 48 h treatment were showed in Supplementary Figure S6. All morphologies of cancer cell lines exhibited varying degrees of alterations, including shrunken cell, arranged loosely and detached from the surface. Ratios of normal morphological cells decreased as the concentration of the extract increased. However, only a few normal colorectal CCD-18Co cells shrank after treatment with the extract. Overall, the extract showed a broad-spectrum antitumor potential against human colon, breast, lung, liver, glioma cancer cells and had no cytotoxicity toward normal human colon cells. Based on the cytotoxicity results, we elected to further characterize signaling events affected by the metabolites of strain 452^T^ in HCT-116, HePG2, and A549 cells.

### Structure and Cytotoxicity of Bioactive Metabolites From Strain 452^T^

**FIGURE 2:**
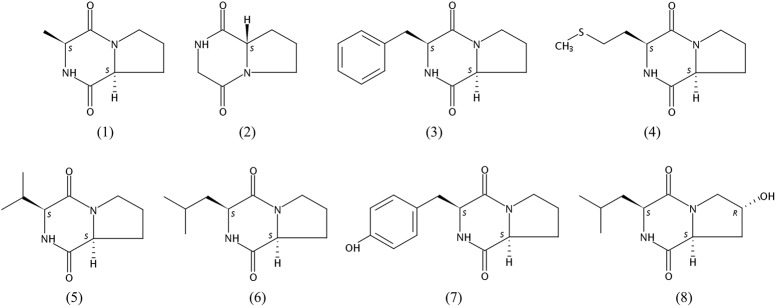
Chemical structures of compounds **1–8**.

Bioassay-guided isolation of the active components of 452^T^ in the ethyl alcohol eluent after a macroporous resin separation yielded eight main bioactive metabolites, which were then characterized by spectroscopic analyses (Supplementary Data Sheet 1) and by comparison with the literature data. As shown in **Figure 2**, the constituents (compounds **1–8**) were identified as cyclo(Pro-Ala) (**1**) (Trigos et al., 1997), cyclo(Pro-Gly) (**2**) (Chen et al., 2012), cyclo(Pro-Phe) (**3**) (Sansinenea et al., 2016), cyclo(Pro-Met) (**4**) (Kumar et al., 2013), cyclo(Pro-Val) (**5**) (Furtado et al., 2005), cyclo(Pro-Leu) (**6**) (Sansinenea et al., 2016), cyclo(Pro-Tyr) (**7**) (Sansinenea et al., 2016), and cyclo(L-Leu-trans-4-hydroxy-L-Pro) (**8**) (Cronan et al., 1998).

The cytotoxic activities of compounds **1–8** against HCT-116, HepG2, and A549 cancer cell lines are shown in **Table 3**. Both compounds **1** and **5** suppressed the proliferation of all tested cells, but compound **1** showed stronger cytotoxic activities than compound **5**. Compounds **1** and **4** exhibited good cytotoxicity against A549 with IC_50_ values of 18.5 and 27.3 μg/mL, compounds **1** and **5** showed cytotoxicity against HCT-116 cells with IC_50_ values of 47.6 and 32.3 μg/mL, which the average IC_50_ values were lower 50.0 μg/mL. The compounds exhibited different degrees of cytotoxicity, compounds **2** and **5–8**, weakly inhibited the growth of some cells (average IC_50_ of 50.0–100.0 μg/mL) or showed no cytotoxicity against some cells (average IC_50_ > 100.0 μg/mL). It is noteworthy that the compounds **1–8** were diketopiperazines which were isolated under guidance of cytotoxic activities. Comparison of the results of the compounds **1–8**, suggested that the proline was probably critical for the cytotoxic activity of diketopiperazine.

**Table 3:** IC_50_ values (μg/mL) of compounds **1–8** against human cancer cell lines.

Metabolite	HCT-116	HepG2	A549
Cyclo(Pro-Ala) (**1**)	47.6 + 2.9	88.3 + 5.6	18.5 + 0.9
Cyclo(Pro-Gly) (**2**)	–	94.5 + 7.3	–
Cyclo(Pro-Phe) (**3**)	32.3 + 1.5	57.0 + 2.1	–
Cyclo(Pro-Met) (**4**)	–	105.5 + 3.2	27.3 + 1.1
Cyclo(Pro-Val) (**5**)	67.2 + 4.9	102.9 + 5.2	111.7 + 2.3
Cyclo(Pro-Leu) (**6**)	92.6 + 7.8	–	120.3 + 6.7
Cyclo(Pro-Tyr) (**7**)	–	84.2 + 8.6	–
Cyclo(L-Leu-trans-4-hydroxy-L-Pro) (**8**)	–	–	92.8 + 2.3
Doxorubicin	1.3 + 0.2	2.9 + 0.3	2.1 + 0.3

*Doxorubicin was tested as a positive control. Values represent the mean of three replications ± standard deviation. –, not tested or average IC_*50*_ > 150.0 μg/mL.*

### GC-MS Analysis of the Extract From Strain 452^T^

**FIGURE 3:**
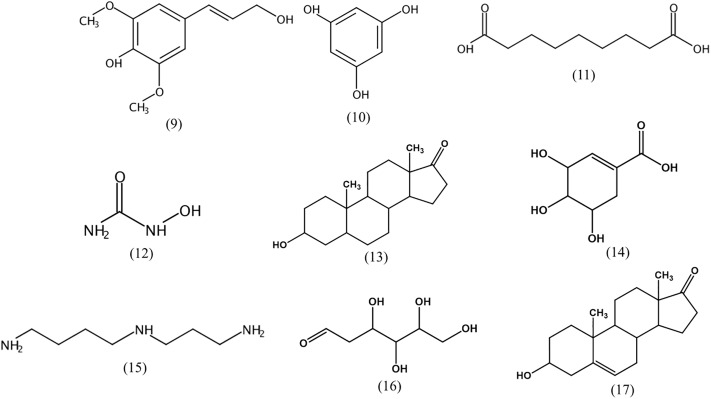
Chemical structures of cytotoxic compounds identified by GC-MS.

*Streptomyces* produce various secondary metabolites with diverse biological activity, and GC-MS analysis is used to facilitate chemical profiling and to identify such active compounds (Ser et al., 2015; Law et al., 2017). GC-MS analysis conducted in the current study revealed the presence of more than 500 compounds in the strain 452^T^ extract, including sinapyl alcohol (**9**), phloroglucinol (**10**), azelaic acid (**11**), hydroxyurea (**12**), androsterone (**13**), shikimic acid (**14**), spermidine (**15**), 2-deoxy-D-glucose (**16**), and dehydroepiandrosterone (**17**) (**Figure 3**). Zhao et al. (2002) isolated seven sinapyl alcohol analogs from *Ligularia nelumbifolia*, and verified their cytotoxic activities against cancer cell lines A549, HL-60, and KB. Phloroglucinol significantly inhibits the growth of a lung carcinoma tumor in the mouse model, and potentially inhibits the bioactivity of endothelial progenitor cells, thereby attenuating tumor growth and angiogenesis (Kwon et al., 2012). Azelaic acid is a natural dicarboxylic acid and an antiproliferative agent that is cytotoxic to a variety of tumor cell lines, including human melanoma cells; because of its inhibition of mitochondrial oxidoreductases of the respiratory chain and of enzymes involved in DNA synthesis, normal cells are unaffected at dosages and times of exposure similar to used for tumor cell treatment (Zaffaroni et al., 1990; Breathnach, 1999). Hydroxyurea is a widely used drug in the therapy for several human cancers; it inhibits deoxynucleotide synthesis and, consequently, DNA synthesis by blocking the cellular enzyme ribonucleotide reductase (Stearns et al., 1963; Lori et al., 1994). Androsterone is an androgen and a steroid hormone drug. Several studies reported that androsterone is cytotoxic to MCF-7 breast cancer cells and HT-29 cancer cells *in vitro* (Carroll et al., 1972; Gao et al., 2010). Dehydroepiandrosterone is a derivative of androsterone, a potent inhibitor of glucose-6-phosphate dehydrogenase, which has been shown to inhibit the growth of neoplasms from the human skin, lung, colon, and mammary tissue (Melvin et al., 1997). Shikimic acid is highly cytotoxic and inhibits the spread of cancer cells in mouse (Hirono et al., 1977; Ganem, 1978). Spermidine is an organic cation required for cell proliferation but its accumulation can induce apoptosis. Spermine triggers the cell death program, characterized by cytochrome c exit from the mitochondria, dATP-dependent processing of pro-caspase-3, and caspase activation (Stefanelli et al., 1999; Zahedi et al., 2007). Finally, 2-deoxy-D-glucose is a glycolysis inhibitor that differentially enhances the radiation and chemotherapeutic drug-induced cell death in cancer cells *in vitro*. Its potential as an immunomodulator, in addition to a direct effect on the tumor, is specifically employed in a combination treatment of Lewis lung carcinoma (Farooque et al., 2014; Pyaskovskaya et al., 2016). The antitumor activity and low toxicity of the above compounds have been documented in numerous studies, supporting the notion that they at least partially, account for the cytotoxic activity of the strain 452^T^ extract.

### LC-MS/MS Analysis of the Extract From Strain 452^T^

**FIGURE 4:**
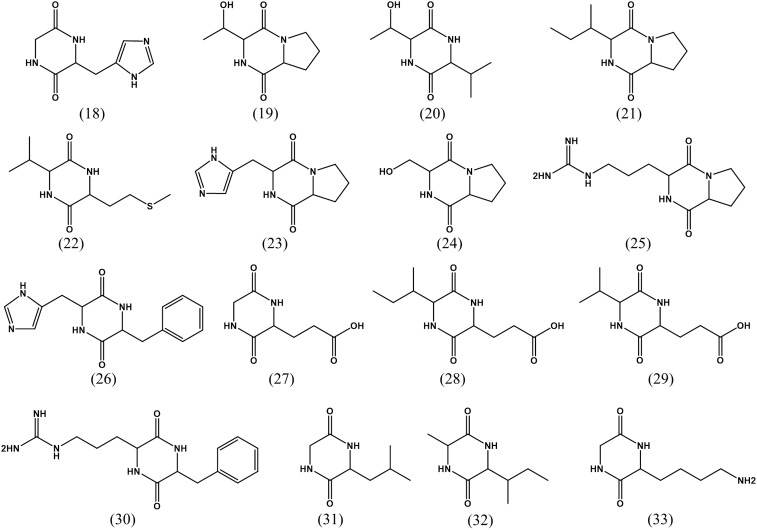
Chemical structures of diketopiperazines indicated by LC-MS/MS.

LC-MS/MS combined with UV data and database analyses are used to confirm the structure of compounds of interest (Ferreira et al., 2016). A variety of diketopiperazines from strain 452^T^ were detected using LC-MS/MS and analyzed by comparing with data in the Human Metabolome Database^5^. Excluding 8 types of diketopiperazine (compounds **1–8**), another 16 types of diketopiperazine were tentatively identified. As shown in **Figure 4**, these were cyclo(Gly-His) (**18**), cyclo(Pro-Thr) (**19**), cyclo(Thr-Val) (**20**), cyclo(Ile-Pro) (**21**), cyclo(Met-Val) (**22**), cyclo(His-Pro) (**23**), cyclo(Pro-Ser) (**24**), cyclo(Pro-Arg) (**25**), cyclo(His-Phe) (**26**), cyclo(Gly-Glu) (**27**), cyclo(Ile-Glu) (**28**), cyclo(Val-Glu) (**29**), cyclo(Arg-Phe) (**30**), cyclo(Gly-Leu) (**31**), cyclo(Ile-Ala) (**32**), and cyclo(Gly-Lys) (**33**). These compounds have been also identified in other microbes.

**FIGURE 5:**
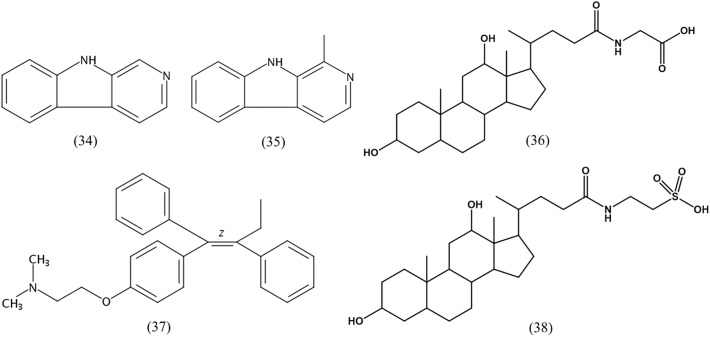
Chemical structures of cytotoxic compounds indicated by LC-MS/MS.

The other detected compounds were β-carboline (**34**), harman (**35**), glycodeoxycholic acid (**36**), tamoxifen (**37**), and taurodeoxycholic acid (**38**) (**Figure 5**). β-Carboline is a natural indole alkaloid, and widely occurs in plants and microbes. Previous studies reported that β-carboline and a series of its derivatives (including harman alkaloids) inhibit DNA topoisomerases and interfere with DNA synthesis; They interact with DNA by groove binding and intercalation, which may lead to major structural changes in the DNA (Nafisi et al., 2010). Further, they exert a pronounced antitumor activity against human tumor cells, including KB, DLD, NCI-H661, Hepa, and HepG2/A2 cell lines (Prinsep et al., 1991; Shen et al., 2011; Silva et al., 2016). Glycodeoxycholic acid and taurodeoxycholic acid are important components of bile acid, and cause liver injury during cholestasis by inducing hepatocyte apoptosis via Fas-dependent and -independent mechanisms (Higuchi et al., 2001). The *p53* gene expression induced telomerase activity and *hTERT* expression was obviously reduced after the human liver cancer cells SMMC7721 were treated by glycodeoxycholic acid, with a subsequent induction of apoptosis of the liver cancer cells (Patel et al., 1994; Toledo et al., 2004). Tamoxifen is an antagonist of the estrogen receptor, able to competitively bind to the estrogen receptor and prevent estrogen activity. It is widely used for breast cancer prevention and treatment, ovarian cancer treatment, and also for the pancreatic cancer and polycystic ovarian syndrome treatment (Abe et al., 2005).

### Description of *Streptomyces nigra* sp. nov.

(*ni’gra*. L. fem. adj. *nigra* black, referring to the color of melanin produced by mature colonies on MA and MB).

Cells are gram-positive, non-motile, and form gray aerial and dark-substrate mycelia on MA. On the ends of the aerial mycelium bud-shaped spores are formed, the spore surface is smooth and there are no spore-chains on aerial mycelium. The colors of the aerial and substrate mycelia are media-dependent. Cells secrete melanin on MA and MB at 28°C, grow well on ISP 2, ISP 3, ISP 4, ISP 5, and ISP 7 agars, and on MA and tryptone soya agar for 7–14 days at 28°C; they grow moderately well on ISP 6 agar, *Streptomyces* agar, starch casein nitrate agar, nutrient agar, and potato-glucose agar. Growth occurs at 15–45°C (25°C optimum), pH 5.0–9.0 (pH 7.0 optimum), and 0–8.0% (w/v) NaCl (1.5% optimum). The cells are positive for catalase, alkaline phosphatase, esterase (C4), esterase lipase (C8), leucine arylamidase, valine arylamidase, trypsin, acid phosphatase, naphthol-AS-BI-phosphohydrolase, *β*-galactosidase, *α*-glucosidase, *β*-glucosidase, *N*-acetyl-*β*-glucosaminidase, and *α*-mannosidase activities; they hydrolyze casein, starch, nitrate, and gelatin; and produce H_2_S. The cells are negative for oxidase, and in methyl red test and Voges-Proskauer test; hydrolysis of L-tyrosine, CM-cellulose, and crystalline cellulose (filter paper); and degradation of Tween 20, 40, 60, and 80. The following compounds are utilized as the sole carbon and energy sources: succinic acid, D-maltose, citric acid, *α*-D-lactose, *myo*-inositol, erythritol, adonitol, D-sorbitol, sucrose, trehalose, saligenin, D-mannose, D-fructose, D-galactose, D-glucose, mannitol, glycerol, L-alanine, L-methionine, L-tryptophan, and L-arginine. The major fatty acids (>5%) are iso-C_16:0_ (31.3%), anteiso-C_15:0_ (16.9%), iso-C_15:0_ (12.5%), C_16:0_ (6.5%), iso-C_16:0_ H (6.1%), anteiso-C_17:0_ (5.7%), iso-C_14:0_ (5.4%), and summed feature 3 (C_16:1_*ω*7*c* and/or C_16:1_*ω*6*c*, 5.2%). The polar lipid profile of strain 452^T^ contains diphosphatidylglycerol, phosphatidylethanolamine, phosphatidylglycerol, phosphatidylinositol, phosphatidylinositol mannoside, three unidentified phospholipids, an unidentified aminolipid, an unidentified phosphoaminolipid, and an unidentified lipid. The DNA G + C content of the type strain is 71.7% mol.

The type strain is 452^T^ (=KCTC 39960^T^ = MCCC 1K03346^T^), which was isolated from the rhizosphere soil of the mangrove *A. marina* forest of Zhangzhou (24°20′, 117°45′) in Fujian Province, China. The GenBank/EMBL/DDBJ accession number for the 16S rRNA gene sequence of strain 452^T^ is MG572975.

## Conclusion

Phylogenetic, genomic, phenotypic, and chemotaxonomic analyses revealed that strain 452^T^ was localized in the clade of the genus *Streptomyces*, but was pronouncedly different from the most closely related type strains. Strain 452^T^ is therefore considered to represent a novel species, for which the name *S. nigra* sp. nov. is proposed. The type strain is 452^T^ (=KCTC 39960^T^= MCCC 1K03346^T^). Treatment with the extract from strain 452^T^ resulted in a 58.5–90.8% inhibition ratio against six types of cancer cell lines when tested at a concentration at 100 μg/mL, indicating a broad-spectrum cytotoxic activity against cancer cells and inactivity toward normal human cells. Hence, strain 452^T^ is potentially a rich reservoir of natural products with antitumor activity.

Bioassay-guided separation and purification on a macroporous resin, silica gel, sephadex LX-20 column, and semi-preparative HPLC were successfully used to identify the active fractions. Further identification by electrospray ionizing-MS and NMR revealed that the active compounds were related to the diketopiperazine family, with different cytotoxic activities against cancer cells, as previously reported and confirmed in the current study (Yang et al., 2002; Tan et al., 2004; Kanoh et al., 2005; Capon et al., 2007). Interestingly, all eight diketopiperazines isolated in the current study contain proline. According to the literature, more than 12 proline-containing diketopiperazines show widespread biological activity, including antitumor activity (Ortiz and Sansinenea, 2017). The array of diketopiperazines produced by strain 452^T^ was investigated by LC-MS/MS, and additional 16 diketopiperazines were detected, and known to possess antitumor activity (Yang et al., 2002; Tan et al., 2004; Kanoh et al., 2005; Capon et al., 2007). Overall, 24 diketopiperazines produced by strain 452^T^ were identified. Chemical analysis via GC-MS and LC-MS/MS further confirmed that the strain produces metabolites with antitumor activity; 14 metabolites previously shown to possess antitumor activity were identified in strain 452^T^ in the current study. In general, compounds **1–8** (eight diketopiperazines) were produced in high enough quantities when the bacterium was grown on MB medium to allow their isolation and identification. Cytotoxicity of these main bioactive metabolites greatly contributed to the observed bioactivity of strain 452^T^, with other metabolites detected in the current study adding to the suppressive effect on tumor cells. This demonstrated the biopharmaceutical potential of the novel *S. nigra* strain to produce bioactive compounds with cytotoxic activity. Future studies should focus on strain 452^T^ as a potentially high-quality and valuable resource for anticancer or chemo-preventive drug discovery.

## Author Contributions

CC designed, carried out the experiments, and wrote the manuscript. YY and RW carried out the experiments. YZ and MW designed the experiments. JW and ZM analyzed experimental results. SCD and CW revised the manuscript.

## Conflict of Interest Statement

The authors declare that the research was conducted in the absence of any commercial or financial relationships that could be construed as a potential conflict of interest.

## Footnotes

1 http://www.bacterio.net/streptomyces.html

2 http://ijs.microbiologyresearch.org

3 http://www.ncbi.nlm.nih.gov

4 https://www.ezbiocloud.net

5 http://www.hmdb.ca
