# Supplementary material for: Streptomyces nigra sp. nov. Is a Novel Actinobacterium Isolated From Mangrove Soil and Exerts a Potent Antitumor Activity in Vitro
Source: Front Microbiol. 2018 Jul 18;9:1587. doi: 10.3389/fmicb.2018.01587 (PMC6058180; doi:10.3389/fmicb.2018.01587)

Fig. S1 Maximum-parsimony phylogenetic tree based on the 16S rRNA gene sequences of strain 452^{T} and representatives of related taxa. Bootstrap values were expressed as a percentage of 1000 replicates and only those higher than 50% were given at the branch points.

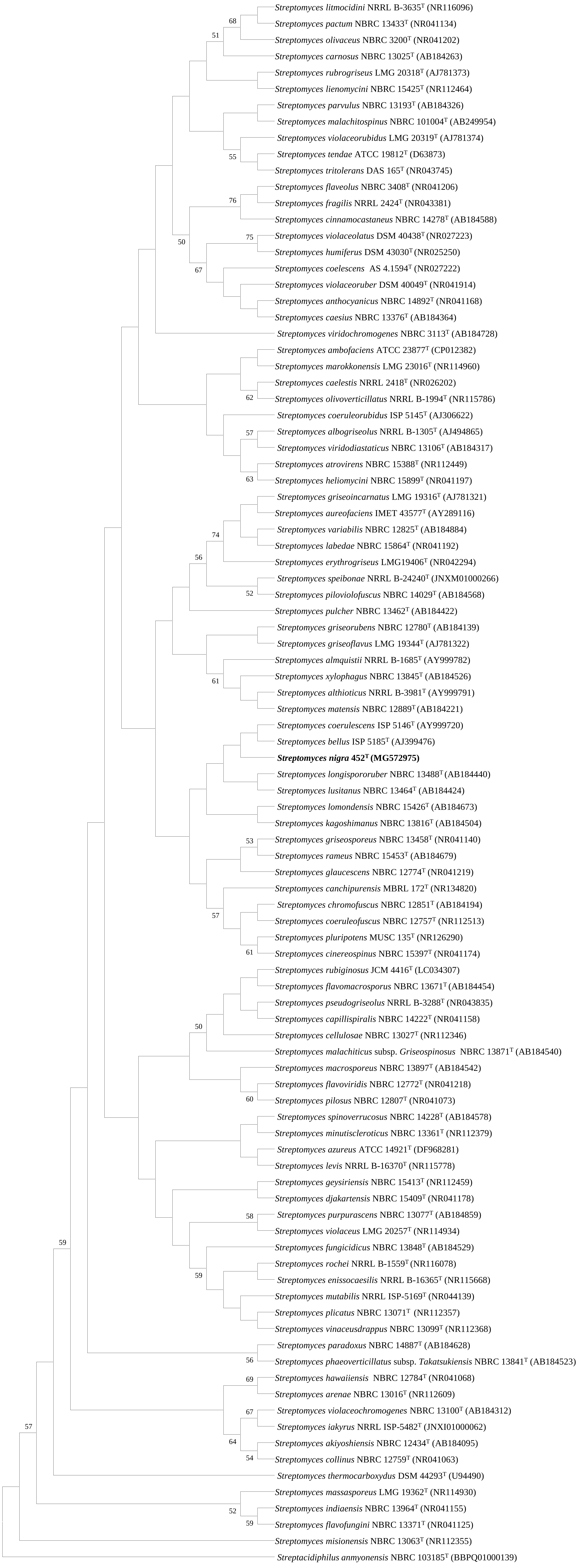

Supplement: Supplementary file 1 [file Image_1.pdf]
