# Supplementary material for: Streptomyces nigra sp. nov. Is a Novel Actinobacterium Isolated From Mangrove Soil and Exerts a Potent Antitumor Activity in Vitro
Source: Front Microbiol. 2018 Jul 18;9:1587. doi: 10.3389/fmicb.2018.01587 (PMC6058180; doi:10.3389/fmicb.2018.01587)

Fig. S3 Transmission electron micrographs (a, b) and scanning electron micrographs (c, d) showing the cell morphology of strain 452^{T}, growing on MA at 28 ℃ for 14 days. Bars represent 5 μm (a, c, d) and 2 μm (b).

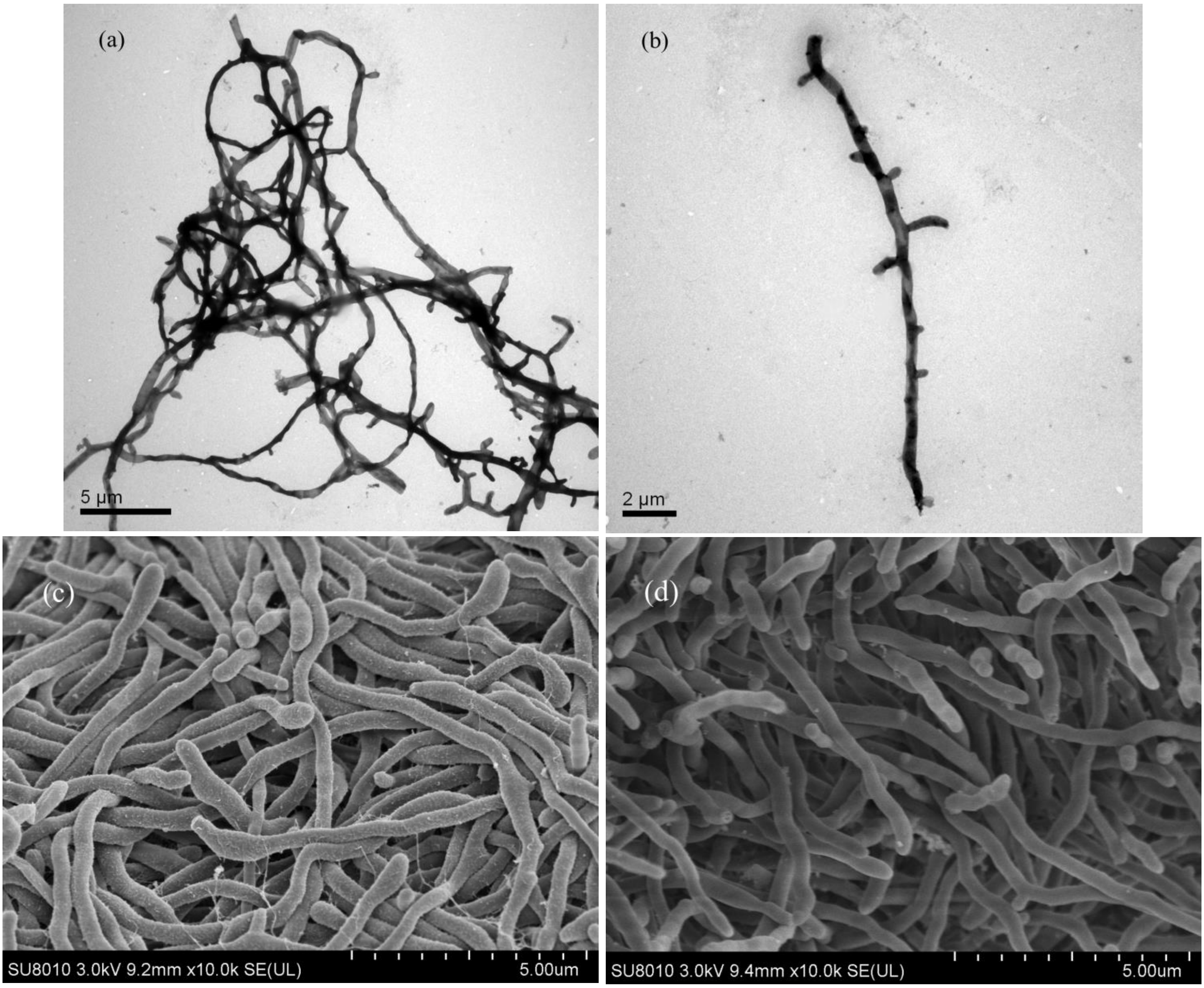

Supplement: Supplementary file 3 [file Image_3.pdf]
