# Supplementary material for: Streptomyces nigra sp. nov. Is a Novel Actinobacterium Isolated From Mangrove Soil and Exerts a Potent Antitumor Activity in Vitro
Source: Front Microbiol. 2018 Jul 18;9:1587. doi: 10.3389/fmicb.2018.01587 (PMC6058180; doi:10.3389/fmicb.2018.01587)

Fig. S4 Polar lipid profile of strain 452^{T}. 1. The plate stained with molybdophosphoric acid; 2. The plate stained with ninhydrin; 3. The plate stained with molybdenum blue; 4. The plates stained with α-naphthol/H_2SO_4. DPG, diphosphatidylglycerol; PE, phosphatidylethanolamine; PG, phosphatidylglycerol; PI, phosphatidylinositol, PIM, phosphatidylinositol mannoside; PL, unidentified phospholipids; AL, unidentified aminolipids; AGL, unidentified phosphoaminolipids; L unidentified lipids.

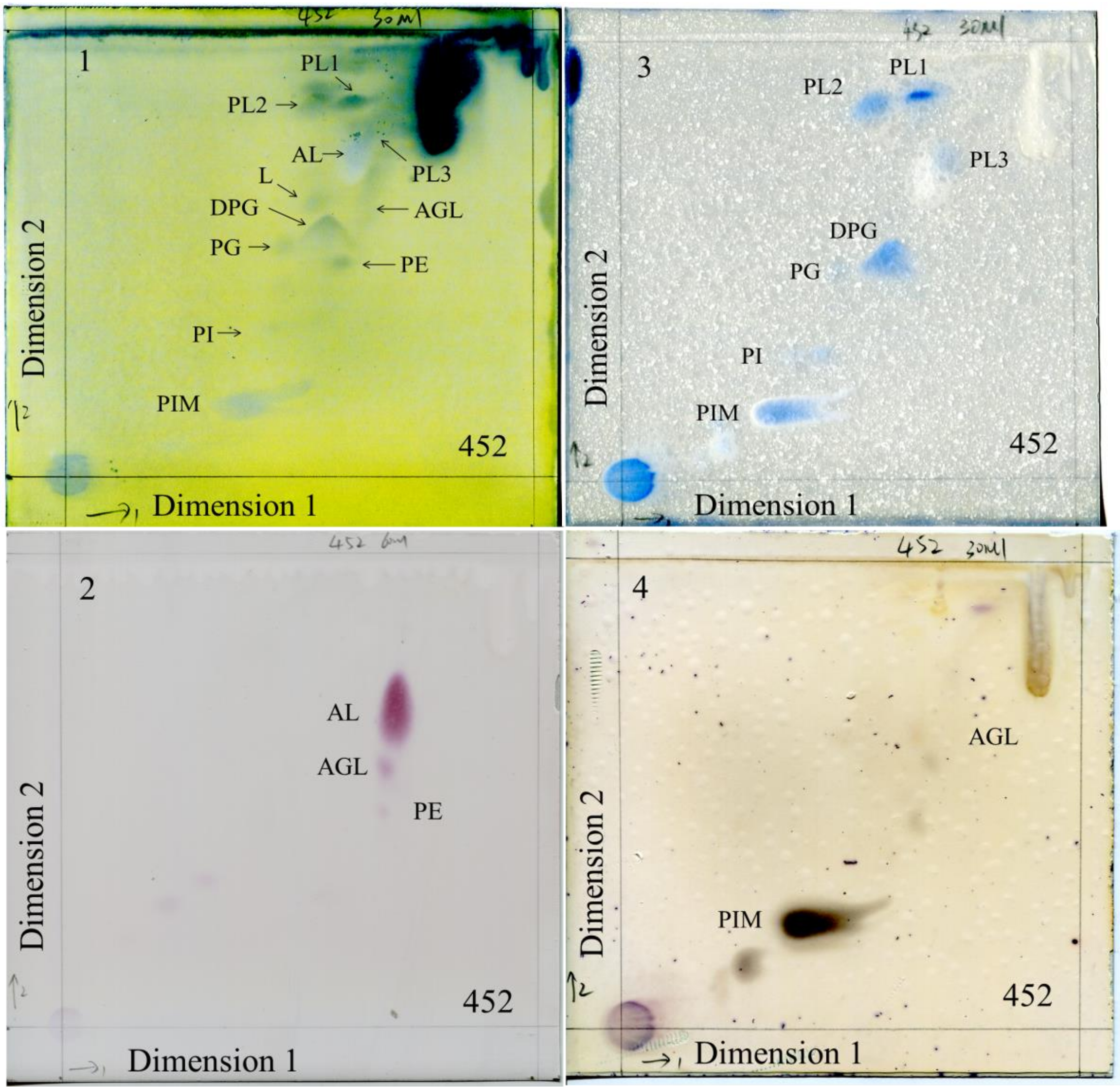

Supplement: Supplementary file 4 [file Image_4.pdf]
