# Supplementary material for: Streptomyces nigra sp. nov. Is a Novel Actinobacterium Isolated From Mangrove Soil and Exerts a Potent Antitumor Activity in Vitro
Source: Front Microbiol. 2018 Jul 18;9:1587. doi: 10.3389/fmicb.2018.01587 (PMC6058180; doi:10.3389/fmicb.2018.01587)

Fig. S5 Cytotoxic activity of 452^T extract *in vitro*. U87, HCT-116, HePG2, A549, MCF-7, and SF268 (all are human cancer cell lines) and CCD-18Co (normal human cell line) were tested. Medium containing 0.5% dimethyl sulfoxide was used as a control. All data are presented as the mean±standard deviation from three experiments.

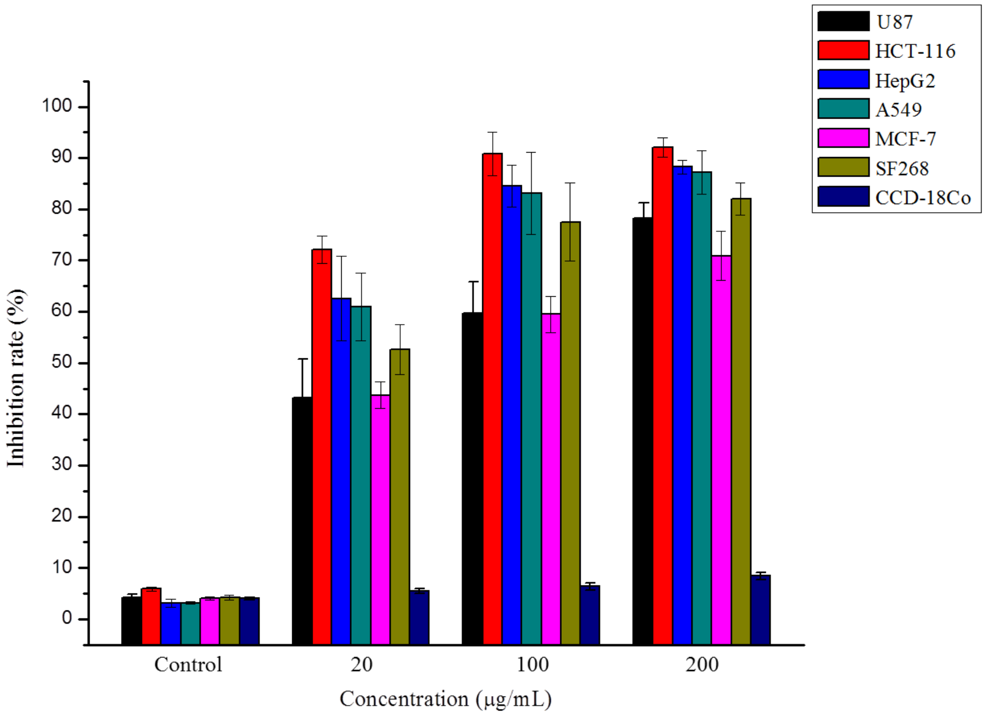

Supplement: Supplementary file 5 [file Image_5.pdf]
