# Supplementary material for: Streptomyces nigra sp. nov. Is a Novel Actinobacterium Isolated From Mangrove Soil and Exerts a Potent Antitumor Activity in Vitro
Source: Front Microbiol. 2018 Jul 18;9:1587. doi: 10.3389/fmicb.2018.01587 (PMC6058180; doi:10.3389/fmicb.2018.01587)
Supplement: Supplementary file 6 [file Image_6.pdf]

Fig. S6 Cytotoxic activity of 452^T extract against human cancer cell lines *in vitro*.
A, Human glioma cancer cell line (U87), B, human colon cancer cell line (HCT-116), C, human liver cancer cell line (HepG2), D, human lung cancer cell line (A549), E, human breast cancer cell line (MCF-7), F, human glioma cancer cell line (SF268), G, human normal colon cell line (CCD-18Co). Cells were treated with negative control (MB with same preparation method of 452^T extract) and 20, 100, 200 μg/mL of the 452^T extract for 48 h, respectively.

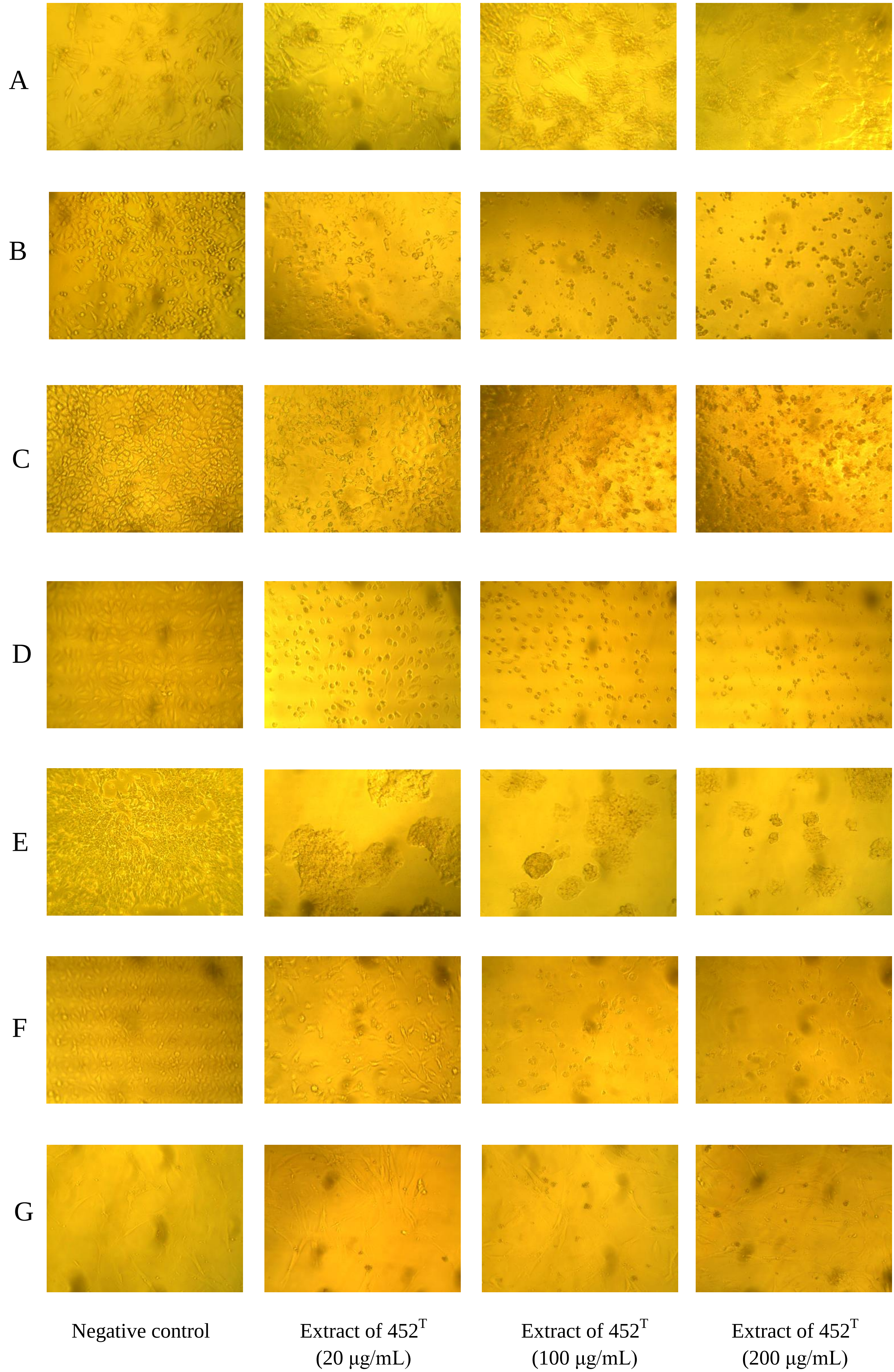
A
B
C
D
E
F
G
Negative control
Extract of 452^{T} (20 μg/mL)
Extract of 452^{T} (100 μg/mL)
Extract of 452^{T} (200 μg/mL)
