# Supplementary material for: Streptomyces nigra sp. nov. Is a Novel Actinobacterium Isolated From Mangrove Soil and Exerts a Potent Antitumor Activity in Vitro
Source: Front Microbiol. 2018 Jul 18;9:1587. doi: 10.3389/fmicb.2018.01587 (PMC6058180; doi:10.3389/fmicb.2018.01587)
Supplement: Supplementary file 8 [file Table_2.pdf]

Table S2. Phenotypic characteristics of strain 452^T and its most closely related species on different mediums.
Taxa: 1, strain 452 ^{T}; 2, *Streptomyces bellus* DSM 40185^T; 3, *Streptomyces coerulescens* DSM 40146^T; 4, *Streptomyces coeruleorubidus* DSM 41172^T. All data were obtained from this study.
-, not detected or no growth

Characteristic		1	2	3	4
ISP2	Colour of aerial	White	White	White	Reddish white
	Colour of substrate	White	White	White	Reddish white
	Diffused pigment	-	-	-	-
	Growth status	Good	Good	Moderate	Good
	Colony surface	Rough	Rough	Smooth	Rough
ISP3	Colour of aerial	Grey	White	White	White
	Colour of substrate	White	White	White	Pink
	Diffused pigment	-	-	-	-
	Growth status	Good	Good	Moderate	Good
	Colony surface	Rough	Rough	Rough	Rough
ISP4	Colour of aerial	Greyish blue	Grey	White	White
	Colour of substrate	White	Brown	White	White
	Diffused pigment	-	-	-	-
	Growth status	Good	Good	Moderate	Moderate
	Colony surface	Hairy	Rough	Hairy	Hairy
ISP5	Colour of aerial	White	White	-	Pink
	Colour of substrate	Yellow	Orange	-	Pink
	Diffused pigment	-	-	-	-
	Growth status	Good	Good	-	Moderate
	Colony surface	Rough	Rough	-	Rough
ISP6	Colour of aerial	Grey	Grey	Reddish brown	Grey
	Colour of substrate	Grey	Grey	Yellowish brown	Grey
	Diffused pigment	Brown	Brown	-	Brown
	Growth status	Moderate	Good	Moderate	Good
	Colony surface	Rough	Smooth	Rough	Smooth
ISP7	Colour of aerial	Grey	Yellowish white	White	Grey
	Colour of substrate	Grey	Yellow	White	Grey
	Diffused pigment	-	-	-	Brown
	Growth status	Good	Good	Moderate	Good
	Colony surface	Smooth	Rough	Hairy	Hairy
NA	Colour of aerial	White	Grey	Yellowish white	Grey
	Colour of substrate	Yellowish brown	White	Yellowish brown	Yellowish brown
	Diffused pigment	-	-	-	-
	Growth status	Moderate	Good	Moderate	Good
	Colony surface	Smooth	Smooth	Rough	Smooth
MA	Colour of aerial	Grey	White	Grey	Brown

	Colour of substrate	Grey	Reddish brown	Grey	Brown
	Diffused pigment	Brown	Brown	Brown	Brown
	Growth status	Good	Good	Moderate	Good
	Colony surface	Rough	Hairy	Rough	Smooth
TSB	Colour of aerial	White	White	White	White
	Colour of substrate	White	White	White	White
	Diffused pigment	-	-	-	-
	Growth status	Good	Good	Moderate	Good
	Colony surface	Rough	Rough	Rough	Rough
SA	Colour of aerial	Yellow	Reddish white	White	Reddish white
	Colour of substrate	White	Yellowish pink	White	Yellowish pink
	Diffused pigment	-	-	-	-
	Growth status	Moderate	Good	Moderate	Good
	Colony surface	Rough	Rough	Hairy	Rough
SCNA	Colour of aerial	Grey	White	White	Reddish white
	Colour of substrate	Yellowish grey	Yellowish pink	Yellowish grey	Yellowish pink
	Diffused pigment	-	-	-	-
	Growth status	Moderate	Good	Moderate	Good
	Colony surface	Hairy	Hairy	Hairy	Hairy
PDA	Colour of aerial	Yellowish white	White	Yellowish white	Reddish white
	Colour of substrate	Yellowish pink	White	Yellowish pink	Reddish white
	Diffused pigment	-	-	-	-
	Growth status	Moderate	Good	Moderate	Good
	Colony surface	Spiny	Hairy	Rough	Rough
