# Supplementary material for: Streptomyces nigra sp. nov. Is a Novel Actinobacterium Isolated From Mangrove Soil and Exerts a Potent Antitumor Activity in Vitro
Source: Front Microbiol. 2018 Jul 18;9:1587. doi: 10.3389/fmicb.2018.01587 (PMC6058180; doi:10.3389/fmicb.2018.01587)
Supplement: Supplementary file 9 [file Table_3.pdf]

Table S3. Fatty acid profiles of strain 452^T and type strains of phylogenetically related species

Strains: 1, strain 452^T; 2, *Streptomyces coeruleorubidus* DSM 41172^T; 3, *Streptomyces bellus* DSM 40185^T; 4, *Streptomyces coerulescens* DSM 40146^T;
-, Not detected; tr, trace amount (<1%).
* Summed features are groups of two or three fatty acids that could not be separated by the MIDI system.
† Summed feature 3 (ECL 15.838) is composed of $C_{16:1}\omega 7c$ and/or $C_{16:1}\omega 6c$.
* Summed feature 5 (ECL 12.759) is composed of $C_{18:2}\omega 6, 9c$ and/or $C_{18:0}$ ante.
‡ Summed feature 9 (ECL 16.443) is composed of $C_{16:0}$ 10-methyl and/or ios-$C_{17:1}\omega 9c$.

**Fatty acids**	**Percentages of total fatty acids**			
	**1**	**2**	**3**	**4**
$C_{14:0}$	tr	tr	1.1	-
$C_{16:0}$	6.5	8.5	12.1	6.5
$C_{17:1}\omega 8c$	-	1.1	tr	2.0
$C_{18:0}$	tr	tr	5.5	1.8
iso-$C_{13:0}$	-	tr	-	1.5
iso-$C_{14:0}$	5.4	1.34	2.1	3.7
iso-$C_{15:0}$	12.5	20.1	15.5	12.4
iso-$C_{16:0}$	31.3	14.1	21.0	14.3
iso-$C_{17:0}$	2.9	8.6	6.8	5.2
iso-$C_{16:0}$ H	6.1	1.9	2.3	5.6
anteiso-$C_{15:0}$	16.9	8.2	8.5	9.4
anteiso-$C_{15:0}\omega 9c$	2.7	2.9	2.1	3.1
anteiso-$C_{17:0}$	5.7	7.0	6.8	6.9
Summed feature 3†	5.2	8.9	4.9	7.2
Summed feature 5*	-	-	1.5	-
Summed feature 9‡	3.2	11.2	7.8	8.1
