# Supplementary material for: Streptomyces nigra sp. nov. Is a Novel Actinobacterium Isolated From Mangrove Soil and Exerts a Potent Antitumor Activity in Vitro
Source: Front Microbiol. 2018 Jul 18;9:1587. doi: 10.3389/fmicb.2018.01587 (PMC6058180; doi:10.3389/fmicb.2018.01587)
Supplement: Supplementary file 10 [file Data_Sheet_1.pdf]

Supplementary materials:
Data of chemical structures of compounds 1-8:

Cyclo (Pro-Ala) **(1)**:

^{1}H NMR (400 MHz, $CDCl_3$) *δ*: 6.45 (1H, s, NH), 4.11 (1H, m, H-6), 4.05 (1H, m, H-3),3.67 (1H, m, H-9a) 3.55 (1H, m, H-9b), 2.43 (1H, m, H-7a), 2.05 (2H, 1H, m, H-7b and H-8a), 1.93 (1H, m, H-8b), 1.51 (3H, d, *J* = 7.2 Hz, H-10); ESIMS (M-H) *m/z* = 167.16. Spectrum recorded obtained in this study matches that reported by Trigos et al. (1997). The constituent was characterized as Cyclo (Pro-Ala).

Cyclo (Pro-Gly) **(2)**:

^{1}H NMR (400 MHz, $CDCl_3$) *δ*: 4.25 (1H,t, *J* = 7.2 Hz, H-6), 4.13 (1H, d, *J* = 16.8 Hz, H-3a), 3.77 (1H, d, *J* = 16.8 Hz, H-3b), 3.55 (2H, m, H-9), 2.34 (1H, m, H-7a), 2.01 (3H, m, H-7b and H-8); ESIMS (M-H) *m/z* = 153.22. Spectrum recorded obtained in this study matches that reported by Chen et al. (2012). The constituent was characterized as Cyclo (Pro- Gly).

Cyclo (Pro-Phe) **(3)**:

^{1}H NMR (400 MHz, $CDCl_3$) *δ*: 7.31 (5H, m, Ph), 5.73 (1H, br s, NH), 4.29 (1H, brd, *J* =10.6, H-3), 4.09 (1H, t, *J* = 7.3 Hz, H-6), 3.67 (1H, m, H-9a), 3.67 (1H, m, H-3), 3.63 (1H, m, H-10a), 2.81 (1H, dd, *J* = 14.4, 10.6 Hz, H-9b), 2.34 (1H, m, H-7a), 2.02 (2H, m, H-7b and H-8a), 1.93 (1H, m, H-8b); ESIMS (M+H) *m/z* = 245.13. Spectrum recorded obtained in this study matches that reported by Sansinenea et al. (2016). The constituent was characterized as Cyclo (Pro-Phe).

Cyclo (Pro-Met) **(4)**:

^{1}H NMR (400 MHz, $CDCl_3$) *δ*: 6.95 (1H, br s, NH), 4.22 (1H, t, *J* = 5.5 Hz, H-3), 4.12 (1H, t, *J* = 8.2 Hz, H-6), 3.62 (1H, m, H-9a), 3.56 (1H, m, H-9b), 2.71 (2H, t, *J* = 6.9 Hz, H-11), 2.41 (1H, m, H-10a), 2.37 (1H, m, H-7a), 2.14 (3H, s, SCH_3), 2.12 (1H, m, H-7b), 2.03 (2H, m, H-10b and H-8a), 1.92 (1H, m, H-8b); ^{13}C NMR (100 MHz,

$CDCl_3$) δ_C: 170.3 (C-4), 165.4 (C-1), 59.0 (C-6), 54.6 (C-3), 45.5 (C-9), 30.2 (C-11), 28.9 (C-10), 28.2 (C-7), 22.7 (C-8), 15.3 (S$\underline{C}H_3$); ESIMS (M+H) *m/z* = 229.00. Spectrum recorded obtained in this study matches that reported by Kumar et al. (2013). The constituent was characterized as Cyclo (Pro-Met).

Cyclo (Pro-Val) **(5)**:

^{1}H NMR (400 MHz, $CDCl_3$) δ: 6.21 (1H, br s, NH), 4.11 (1H, m, H-6), 3.95 (1H, br. s H-3), 3.66 (1H, m, H-9a), 3.55 (1H, m, H-9b), 2.65 (1H, m, H-7a), 2.40 (1H, m, H-7b), 2.06 (2H, m, H-8), 1.93 (1H, m, H-10), 1.07 (3H, d, J = 7.1 Hz, H-11), 0.92 (3H, d, J = 6.8 Hz, H-12); ESIMS (M+H) *m/z* = 197.19, ESIMS (M-H) *m/z* = 195.14. Spectrum recorded obtained in this study matches that reported by Furtado et al. (2005). The constituent was characterized as Cyclo (Pro-Val).

Cyclo (Pro-Leu) **(6)**:

^{1}H NMR (400 MHz, $CDCl_3$) δ: 6.46 (1H, br s, NH), 4.10 (1H, t, J = 8.0 Hz, H-6), 3.94 (1H, m, H-3), 3.67 (1H, m, H-9a), 3.54 (1H, m, H-9b), 2.42 (1H, m, H-5a), 2.06 (1H, m, H-5b), 2.03 (2H, m, H-10a and H-8a), 1.92 (1H, m, H-8b), 1.78 (1H, m, H-11), 1.67 (1H, m, H-10b), 1.01 (3H, d, J=6.4 Hz, H-12), 0.97 (3H, d, J = 6.3 Hz, H-13); ESIMS (M-H) *m/z* = 209.21. Spectrum recorded obtained in this study matches that reported by Sansinenea et al. (2016). The constituent was characterized as Cyclo (Pro-Leu).

Cyclo (Pro-Tyr) **(7)**:

^{1}H NMR (400 MHz, CD_3OD) δ: 7.00 (2H, d, J = 8.2 Hz, H-2’, H-6’), 6.74 (2H, d, H-3’, H-5’), 4.24-4.32 (1H, m, H-3), 4.16 (1H, t, J = 4.2 Hz, H-6), 3.55 (2H, m, H-7), 3.13 (1H, dd, J = 13.8, 4.2 Hz, H-10a), 2.89 (1H, dd, J = 13.8, 4.5 Hz, H-10b), 2.64 (1H, m, H-7a), 2.08 (1H, m, H-8a), 1.93 (1H, m, H-7b), 1.68 (1H, m, H-8b); ESIMS (M+H) *m/z* = 261.11. Spectrum recorded obtained in this study matches that reported by Sansinenea et al. (2016). The constituent was characterized as Cyclo (Pro-Tyr).

Cyclo (L-leu-trans-4-hydroxy-L-pro) **(8)**:

^{1}H NMR (400 MHz, $CDCl_3$) δ: 6.10 (1H, s, NH), 4.59 (1H, brs, H-8), 4.51 (1H, dd, J = 10.8, 6.6 Hz, H-3), 4.07 (1H, brd, J = 8.3 Hz, H-6), 3.72 (1H, dd, J = 13.2, 3.8 Hz, H-9a), 3.58 (1H, d, J = 13.2 Hz, H-9b), 2.40 (1H, m, H-7a), 2.15 (1H, m, H-7b), 2.06 (1H, m, H-10a), 1.77 (1H, m, H-11), 1.54 (1H, m, H-10b), 1.01 (3H, d, J = 6.5 Hz, H-12), 0.98 (3H, d, J = 6.3, H-13); ^{13}C NMR (100 MHz, $CDCl_3$) δ_C: 170.5 (C-4), 166.2 (C-1), 68.4 (C-8), 57.4 (C-3), 54.4 (C-9), 53.4 (C-6), 38.6 (C-10), 37.4 (C-7), 24.7 (C-11), 23.2 (C-12), 21.3 (C-13). ESIMS (M-H) *m/z* = 225.14. Spectrum recorded obtained in this study matches that reported by Jr et al. (1998). The constituent was characterized as Cyclo (L-leu-trans-4-hydroxy-L-pro).

Reference:

Chen, J.H., Lan, X.P., Liu, Y.H., and Jia, A.Q. (2012). The effects of diketopiperazines from Callyspongia sp on release of cytokines and chemokines in cultured J774A.1 macrophages. *Bioorganic & Medicinal Chemistry Letters* 22(9)**,** 3177-3180. doi: 10.1016/j.bmcl.2012.03.045.

Furtado, Pupo, N.A.J.C., T.Carvalho, M.n., IvoneCampo, Duarte, V.L., Bastos, M.C.T., et al. (2005). Diketopiperazines produced by an Aspergillus fumigatus Brazilian strain. *J.braz.chem.soc* 16(6)**,** 1448-1453.

Jr, J.M.C., Davidson, T.R., and Singleton, F.L. (1998). Plant Growth Promoters Isolated from a Marine Bacterium Associated with Palythoa sp. *Natural Product Letters* 11(4)**,** 271-278.

Kumar, N., Mohandas, C., Nambisan, B., Kumar, D.R., and Lankalapalli, R.S. (2013). Isolation of proline-based cyclic dipeptides from Bacillus sp. N strain associated with rhabditid [corrected] entomopathogenic nematode and its antimicrobial properties. *World Journal of Microbiology & Biotechnology* 29(2)**,** 355-364.

Sansinenea, E., Salazar, F., Jimenez, J., Mendoza, A., and Ortiz, A. (2016). Diketopiperazines derivatives isolated from Bacillus thuringiensis and Bacillus endophyticus, establishment of their configuration by X-ray and their synthesis. *Tetrahedron Letters* 57(24)**,** 2604-2607. doi: 10.1016/j.tetlet.2016.04.117.

Trigos, A., Reyna, S., Gutierrez, M.L., and Sanchez, M. (1997). Diketopiperazines from Cultures of the Fungus Colletotrichum gloesporoides. *Natural Product Letters* 11(1)**,** 13-16.
